# Hearing impairment after acute bacterial meningitis in children

**DOI:** 10.12669/pjms.343.14373

**Published:** 2018

**Authors:** Fatima Zeeshan, Attia Bari, Mubeen Nazar Dugal, Fauzia Saeed

**Affiliations:** 1 Dr. Fatima Zeeshan, MRCPCH (UK), FCPS. Department of Paediatric Medicine, The Children’s Hospital and The Institute of Child Health, Lahore, Pakistan; 2 Dr. Attia Bari, DCH, MCPS, FCPS, MHPE. Department of Paediatric Medicine, The Children’s Hospital and The Institute of Child Health, Lahore, Pakistan; 3 Dr. Mubeen Nazar Dugal, FCPS Department of Paediatric Medicine,. The Children’s Hospital and The Institute of Child Health, Lahore, Pakistan; 4 Dr. Fauzia Saeed, MSC Audiology, Department of Audiology, The Children’s Hospital and The Institute of Child Health, Lahore, Pakistan

**Keywords:** Otoacoustic emissions, Sensorineural hearing loss, Tympanogram

## Abstract

**Objective:**

To determine the incidence of hearing loss after acute episode of meningitis in children

**Methods:**

A descriptive study carried out in the Department of Pediatric Medicine of The Children’s Hospital Lahore, Pakistan from January 2014 to July 2016. A total of 175 children one month to 13 years of age admitted with diagnosis of meningitis were included. Complete blood count, CSF cytology, biochemistry and culture sensitivity were sent. CT scan brain was done if required. Hearing assessment was done two weeks after admission using otoacoustic emissions in the patients having normal tympanogram. Hearing impairment was classified as sensorineural if otoacoustic emissions were absent while tympanometry was normal.

**Results:**

Of 175 children, 58% were males and 42% were females. Mean age was 2.1 years. Orientation as assessed by Glasgow comma scale (GCS) was normal in 63% while 5% had GCS<8 and 32% had GCS between 8 and 15. Signs of meningeal irritation were seen in 58% while focal signs only in 4%. In 15 % cases CT scan was done, out of which 73% showed abnormal findings. Otoacoustic emissions were absent in 22% of cases. Risk factors of hearing deficit were stay duration of more than 10 days (p=0.04), low GCS at presentation (p=0.009) and meningitis with complications (p=0.008).

**Conclusion:**

The frequency of hearing loss is 22% following acute episode of meningitis which necessitates the need for implementation of screening assessment after meningitis in Pakistan. Prolonged stay, low GCS and complicated meningitis are risk factors for hearing impairment.

## INTRODUCTION

Hearing deficit is a major complication of meningitis in children.^1^ Hearing loss is estimated to be 10% in developed countries whereas transient hearing loss occurs much more frequently. More than half of acquired cases of deafness are due to meningitis.^2^ It has been observed that this occurs in the initial phase of illness.^3^ Severity of meningitis and presence of comorbidity are important risk factors.^4^ Sensorineural hearing loss is found to be highly prevalent in children suffering from meningitis which undermines the importance of proper and timely audiological testing.^1^

In Pakistan, there are very few studies done on assessment hearing loss in such children. One study yielded hearing loss incidence to be 18.5%.^5^ Since bacterial meningitis is a very serious and common presentation of children at emergencies of big pediatric centers in our country so we decided to find the spectrum of hearing loss in children after acute meningitis and its relation to age, sex, altered sensorium, cerebrospinal fluid (CSF) findings, stay duration, treatment and complications.

## METHODS

This was a hospital based descriptive cross sectional study, conducted at The Children’s Hospital and The Institute of Child Health Lahore. A total of 175 children one month to 13 years of age admitted with diagnosis of acute bacterial meningitis were included. Positive CSF was defined as white blood cell (WBC) more than 10/ul in CSF. Children with meningomyelocele, prior central nervous system pathology, seizure disorder, hydrocephalus, history of recurrent meningitis and acute head trauma were excluded.

A detailed history and thorough clinical examination was undertaken for each patient. Their clinical features focusing mainly age, gender, presenting symptoms, examination findings, duration of illness before presentation, total hospital stay, complications and outcome were documented. The fontanelle was examined for children less than two years. In all patients’ relevant laboratory data including complete blood count (CBC), CSF cell count with differential, protein, glucose, gram staining with CSF culture sensitivity (c/s) was performed. The patients were managed with intravenous antibiotics. Dexamethasone was given at a dosage of 0.6 mg/kg/day at onset within half hour of the first dose of antibiotic above four months of age as per hospital protocol. Informed consent was obtained from parents before enrolling children into the study. The ethical approval was taken from local hospital committee. The antibiotics were continued for a minimum of 10 days and were switched to second line according to CSF C/S report thus treatment was individualized based on each patient’s clinical course. All patients were examined daily for neurological complications such as seizures after four days of treatment, motor and sensory loss and cranial nerve palsy. CT scan brain was used when required and results were documented.

Hearing assessment was performed after two weeks of presentation with meningitis using otoacoustic emissions. First tympanometry was done to rule out conductive deafness. Those patients who had abnormal tympanogram were not subjected to otoacoustic test. Those with abnormal tympanogram were sent to ENT for further evaluation and scheduled for follow up later. Hearing impairment was classified as sensorineural if otoacoustic emissions were absent in the presence of normal tympanometry.

We used standard deviation for continuous variables and frequency percentages for categorical variables. The Chi Square was applied where applicable. The level of significant was considered p < 0.05.

## RESULTS

We analyzed outcomes of 175 children admitted with diagnosis of acute bacterial meningitis. Of 175 children 102 (58%) were males and 73 (42%) were females. Majority of children were less than five years i.e. 138(79%) whereas only 37 (21%) children were more than ten years of age. Mean age was 2.1 years. Total stay was more than ten days in 66 (38%) cases while 109 (62%) had stay less than ten days.

Nearly all cases i.e. 173 children (99%) presented with fever followed by seizures which were present in 155 (89%) children. We found 9(5%) children had GCS<8 while 56 (32%) children had GCS between 8 and 15 while 110 (63%) had normal score. Signs of meningeal irritation were seen in 101 (58%) while focal signs were observed only in 7(4%) and irritability was seen in 38% cases.CSF count was raised more than 100 cells in 45 (27%) cases while 92 (53%) patients had between 50-100 cells and 37 (20%) had between 10-50 cells. Neutrophil predominance in CSF was seen in 93% cases. Abnormal glucose was seen in 123 (70%) patients while CSF protein was increased in 76 patients (43%).

**Fig.1:**
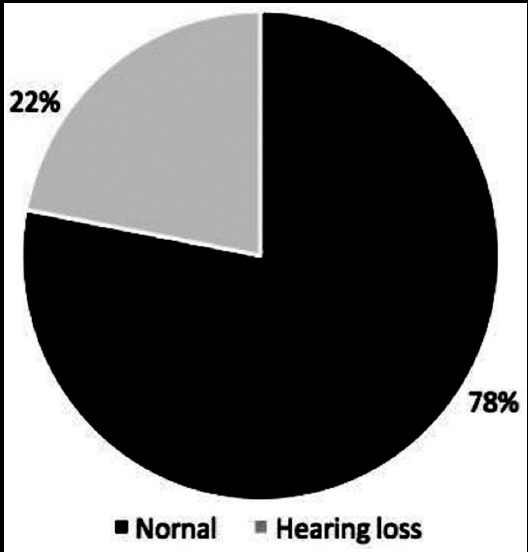
Hearing loss after meningitis

**Fig.2:**
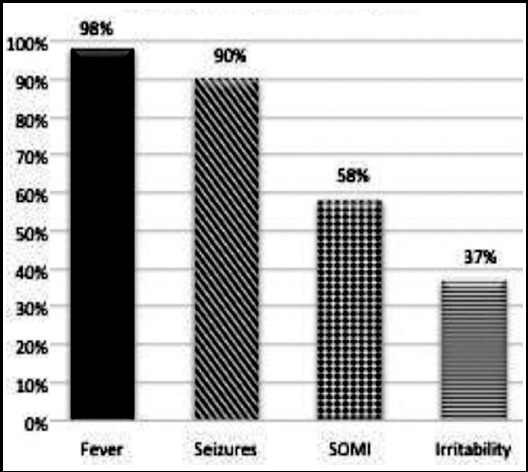
Clinical Presentation.

CSF culture was positive in 11 (6%) patients while complications were seen in 16 (9%) patients. The most frequent early clinical complication was focal deficit in 7 (4%) patients followed by hydrocephalus in 7 (4%) and one each of cranial nerve palsy, and visual loss. The duration of illness before presentation was less than two days in half of cases whereas 21(12%) had illness lasting for more than five days and 66 (38%) had between two and five days. The frequency of clinical parameters assessed is given (Fig.1). CT scan brain was done in 26 children (15%) out of which 19 (11%) cases showed abnormal findings (Table-I). The most common CT finding documented was subdural effusion in 12 patients followed by hydrocephalus in four then hemorrhagic infarcts in two and focal changes in one. CSF culture was positive in 11 children (6%) who showed *Klebsiella* in 8 (4.5%) children followed by *Streptococcus pneumonia* in 2(1%) patients and then coagulase negative *staphylococcus* in one (0.5%). Hearing test was abnormal in 38 (22%) children (Fig.2).

**Table-I:** Relationship of risk factors with hearing loss.

Variables	Percentages n=175	p-values
*Fever*		
Yes	173(99%)	0.4
No	2(1%)	
*Sex of* patient		
Male	102(58%)	0.9
Female	73(42%)	
*Stay duration*		
<10days	109(62%)	0.04
>10 days	66(32%)	
*Seizures*		
Yes	155(89%)	0.9
No	20(11%)	
*Complications*		
Yes	17(9%)	0.008
No	158(91%)	
*CT scan brain result*		
Normal	19 (75%)	0.2
Abnormal	7 (25%)	
*GCS*		
<8	12(7%)	0.009
8-15	56(32%)	

Hearing deficit was correlated with stay duration of more than 10 days (p=0.04), low GCS at presentation (p=0.009) and meningitis with complications (p=0.008) (Table-I). Illness prior to presentation, age, CSF cell counts and culture had no statistical bearing on hearing loss. There was no case fatalities observed.

## DISCUSSION

Bacterial meningitis is the most common cause of acquired hearing loss in childhood.^2^ The exact mechanism is unknown but the cause is thought to be insult to the cochlea and labyrinthine system. The best test is tympanometry and otoacoustic emissions followed by auditory brainstem reponse.^6^ Rates of sensorineural hearing loss varying from 5 to 35% have been seen in American studies with 4% having profound deafness. Hearing can have a significant impact on child’s speech especially if child is preverbal and on learning and social development so this complication should be detected and addressed timely.

There have been very few studies in Pakistan on hearing deficit, one study in 2008 showed nearly similar frequency of 20%as ours.^7^ Some studies report incidence of 30%^8^ whereas a study in Kenya showed incidence of 43%.^8^ Where as a UK study shows only 7.4% meningitis cases with hearing loss.^9^ The increased hearing loss in developing countries can be due to incomplete vaccination and complicated course of meningitis. In another cohort study of six years in Netherlands hearing loss was found to be13%.^10^

Comparable with other studies gender had no relationship with hearing loss in our data.^1^ Walter et al. showed male gender as an independent risk factor of hearing loss.^8^ Earlier age of onset less than 12 months also led to hearing loss as previously reported.^11^

The classic triad of fever, fits and signs of meningeal irritation (SOMI) are present in published literature in 50% whereas focal signs in 33% and unconsciousness in 14%.^12^ In our study SOMI were seen in 102 (58%) and focal signs in 7 (4%) whereas 64 (37%) had altered sensorium. In another study neck stiffness was seen in 83% cases and altered sensorium in 69%.^13^,^14^

In our study the duration of illness before presentation was two days which was similar to other studies which suggest that parents approached health care facility within two days. Most studies show that the incidence of hearing loss is higher in children who were more toxic looking and sick^14^ and we also found this association in children with complicated meningitis (p=0.008).

Hib immunization has eliminated almost all the cases of meningitis caused by *Haemophilus influenza B* whereas *Streptococcus Pneumonia* and *Neisseria meningitis* are common pathogens leading to meningitis. In another study *Streptococcus Pneumonia*ca used 22% where as *Neisseria* caused 8% of loss.^13^ In our study the CSF culture positivity was 6% and out of this 4.5% were gram negative including *Klebsiella* followed by *Streptococcus pneumonia* 1% and coagulase negative *staph aureus* 0.5%. In a recent study of hearing loss in UK *Meningococcal* is isolated in 75% followed by *Streptococcus pneumonia* in 15%cases.^14^ The low yield in our study could be due to faulty culture techniques and prior undocumented antibiotic exposures. In a study of 199 children CSF culture positivity was 12%%.^15^

Otoacoustic emission is a noninvasive test which gives flection of functioning of inner ear status and has been used as a screening. It can be affected by middle ear infections. Only patients with normal tympanogram were subjected to otoacoustic emission test. It cannot reflect function of auditory pathways so its presence cannot rule out hearing loss.^16^ It cannot grade the loss so we could not categorize our patients into those having mild, moderate or severe loss.^17^

We found hearing deficit was strongly correlated with prolonged stay more than 10 days, low GCS and complicated meningitis (Table-I). Complications especially cranial nerve palsy and prolonged stay was also considered risk factor in other studies.^8^ Poor GCS indicates complications and it is linked to neurological complications of hearing deficit as in our study.^18^,^19^ The number of days of illness persisted before presenting to hospital and type of treatment had no impact on hearing deficit in our study like previously mentioned studies.^20^

### Limitations

We could not grade the severity of hearing loss because otoacoustic emission is a screening test which represents cochlear function itself and needs more specialized testing to confirm it. We could not perform Auditory Brainstem Response due to our technical limitations so we could only identify the patients at risk of deafness.

## CONCLUSION

Keeping in view the high rate of occurrence of hearing loss after meningitis in pediatric population in our study we believe the universal hearing screening protocol after meningitis should be implemented in Pakistan. It will help detect early hearing deficit and timely intervention can be implemented to reduce the social and educational challenges for the affected children.

### Author`s contribution

**FZ:** Main Author designed and did statistical analysis & editing along with writing of manuscript.

**AB:** Did critical review & final edit.

**MN and FS:** Involved in methadology & lab investigaitons.
